# Migrating a research data warehouse to a public cloud: challenges and opportunities

**DOI:** 10.1093/jamia/ocab278

**Published:** 2021-12-17

**Authors:** Michael G Kahn, Joyce Y Mui, Michael J Ames, Anoop K Yamsani, Nikita Pozdeyev, Nicholas Rafaels, Ian M Brooks

**Affiliations:** 1 Section of Informatics and Data Science, Department of Pediatrics, University of Colorado School of Medicine, Aurora, CO, USA; 2 Colorado Center for Personalized Medicine, University of Colorado School of Medicine, Aurora, CO, USA; 3 SADA, Inc., North Hollywood, CA, USA; 4 Division of Endocrinology, Metabolism and Diabetes, University of Colorado School of Medicine, Aurora, CO, USA; 5 Division of Biomedical Informatics and Personalized Medicine, Department of Medicine, University of Colorado, Aurora, CO, USA

**Keywords:** data warehousing, cloud computing, big data, research data governance

## Abstract

**Objective:**

Clinical research data warehouses (RDWs) linked to genomic pipelines and open data archives are being created to support innovative, complex data-driven discoveries. The computing and storage needs of these research environments may quickly exceed the capacity of on-premises systems. New RDWs are migrating to cloud platforms for the scalability and flexibility needed to meet these challenges. We describe our experience in migrating a multi-institutional RDW to a public cloud.

**Materials and Methods:**

This study is descriptive. Primary materials included internal and public presentations before and after the transition, analysis documents, and actual billing records. Findings were aggregated into topical categories.

**Results:**

Eight categories of migration issues were identified. Unanticipated challenges included legacy system limitations; network, computing, and storage architectures that realize performance and cost benefits in the face of hyper-innovation, complex security reviews and approvals, and limited cloud consulting expertise.

**Discussion:**

Cloud architectures enable previously unavailable capabilities, but numerous pitfalls can impede realizing the full benefits of a cloud environment. Rapid changes in cloud capabilities can quickly obsolete existing architectures and associated institutional policies. Touchpoints with on-premise networks and systems can add unforeseen complexity. Governance, resource management, and cost oversight are critical to allow rapid innovation while minimizing wasted resources and unnecessary costs.

**Conclusions:**

Migrating our RDW to the cloud has enabled capabilities and innovations that would not have been possible with an on-premises environment. Notwithstanding the challenges of managing cloud resources, the resulting RDW capabilities have been highly positive to our institution, research community, and partners.

## BACKGROUND AND SIGNIFICANCE

Data-intensive programs in personalized medicine, learning health systems, and data-driven research have driven explosive growth in clinical research databases. Research data warehouses (RDWs) now store data from electronic health records; clinical images, videos, and physiological signals; genomic panels expanding to whole-exome/whole-genome sequences; and patient-generated data from mobile apps, home monitors, and wearable devices.^1^^,^^2^ Many RDWs also integrate nonclinical data such as social media postings and public data sets (census, environmental, traffic, crime).^3–8^ The volume of relevant electronic data and the computational requirements to perform advanced analytics using these data easily overwhelm the computing resources of large and small research organizations. Many healthcare organizations are investigating migrating research computing systems from on-premises, locally managed environments to public clouds. We describe the University of Colorado Anschutz Medical Campus’s (CU-AMC) experience implementing a large RDW combining administrative, clinical, genomic, and population-level data from 4 organizations plus commercial, governmental, and public third-party data sources into Google Cloud Platform (Google Cloud Platform). Public cloud providers offer different, rapidly evolving technologies; however, lessons learned from our implementation should apply to organizations considering transitioning to any public cloud provider.

In August 2013, CU-AMC established a new research data warehouse called Health Data Compass (HDC: www.healthdatacompass.org) in partnership with 2 affiliated health systems and an independent faculty practice plan. After an extensive market search, in July 2014, HDC began implementing an on-premises, vendor-supported, healthcare-specific hardware and software stack that included a specialized database engine and extraction-transform-load (ETL) pipeline. Full-scale go-live commenced March 2015.

By summer 2015, despite being just over 1 year into implementation and 3 months into deployment, substantial HDC personnel time and consulting costs were being consumed reacting to unexpected system failures due to computational and storage constraints. Frequent upgrades and patches would take the entire HDC environment offline for more than 24 h. Loading data from hospital sources and running the vendor-provided master person index also overloaded on-premises resources, causing frequent delays in loading new data. In addition, HDC faced unplanned costs to implement redundant hardware and software to support system reliability and disaster recovery. Because of these challenges, HDC initiated 2 formal pilot studies using GCP from April through October 2016. Study 1 targeted high-throughput data processing; study 2 focused on computational scalability. Both pilots also provided insights into technical effort, data security, regulatory compliance, and estimated immediate and long-term costs. Based on these findings, in November 2016, the HDC executive sponsors approved reimplementing the HDC environment within GCP. The transition to a cloud-only infrastructure was completed by February 2017. In late 2020, CU-AMC’s on-premises high-performance compute cluster (HPCC), used for large-scale genomic analyses, reached end-of-life. All next-generation bioinformatics computing and storage needs were migrated to Compass GCP.

Much literature, mostly from marketing or consulting sources, highlights the benefits of cloud-based infrastructures.^9–11^ Generalized guidance for on-premises to in-cloud transitions are harder to find. Given the nascent state of clinical data warehousing in the cloud in late 2016, there was minimal literature and hands-on experience with cloud implementations. Our journey as early cloud adopters provides useful insight into developing a cloud healthcare ecosystem, with emphasis on the additional requirements of a clinical research environment.

## OBJECTIVE

We present the key objectives that HDC articulated at the beginning of the migration from an existing on-premises RDW to a de novo cloud-based reimplementation. We share insights that may be useful to others considering a cloud-based research data warehouse. We also provide usage and cost metrics and describe examples where design decisions can significantly impact the overall costs of a cloud-based deployment.

## MATERIALS AND METHODS

This is a descriptive study. Primary materials date from early 2016 to early 2021, including internal and public presentations before and after the transition, analysis documents, and historical billing records. Costs were aggregated over time and by GCP service. Not included were internal personnel and external consulting costs, although staffing considerations are discussed. Findings were aggregated into topical categories such as networking and security, computation, storage, and staffing. Key performance indicators were generated in June 2021.

## RESULTS

**Figure 1.**
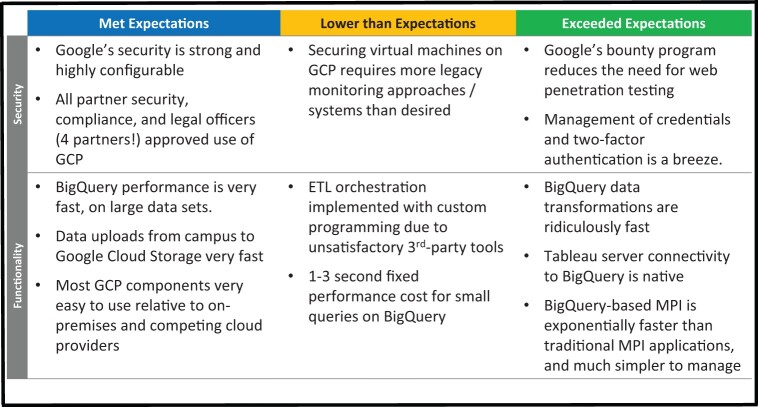
Key findings from 2016 pilot studies comparing Google Cloud Platform with existing on-premises systems as presented to nontechnical executive sponsors. Superlative were used to emphasize particularly distinctive findings that supported the migration proposal.

Figure  1 is the graphic that summarized the findings from the 2016 GCP pilot studies. Findings were separated into “met expectations,” “lower than expectations,” and “exceeded expectations.” Assessments were qualitative except for financial projections. Through the intervening 5 years, these 2016 findings have been re-confirmed. Additional findings have emerged as HDC’s size and functionality expanded from pilot to enterprise-scale.

**Figure 2.**
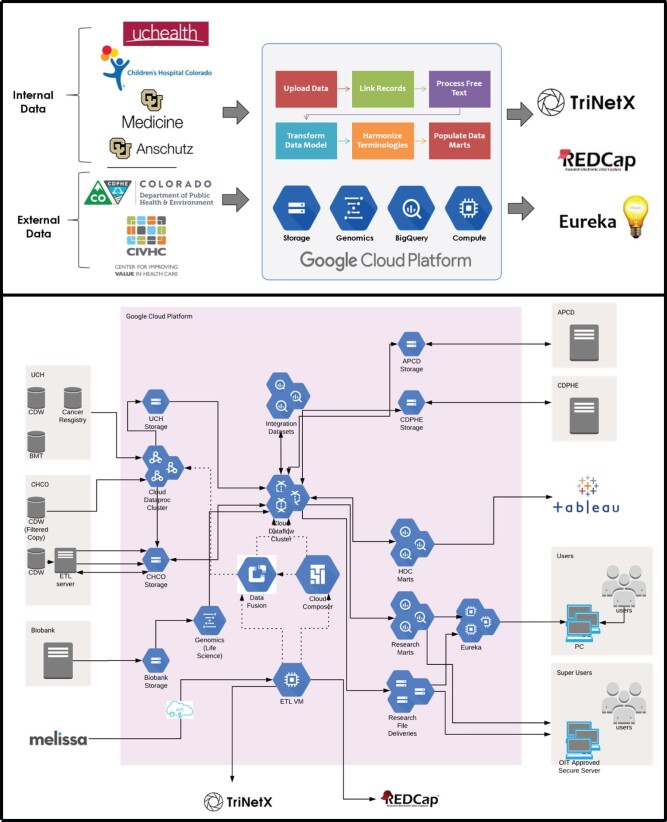
Top, The executive view of Health Data Compass highlighting data inputs, outputs and key GCP technologies for nontechnical audiences. Bottom, Technical view of data flows, network boundaries, and internal GCP technologies used in the current Health Data Compass research data warehouse. Google Cloud icons labels available at https://docs.google.com/presentation/d/1aGOTpNdCoO4GXZ2es38ZFO5qPGEAjTtDSVeHaDpwsas/edit#slide=id.g5e923c6224_190_56. Abbreviations: APCD: Colorado All Payers Claims Database; CDPHE: State death registry; GCP: Google Cloud Platform; Melissa: Melissa Inc.

Figure  2 (top) is the high-level description of HDC’s current capabilities as presented to nontechnical executive audiences. Figure  2 (bottom) is a technical overview that illustrates data flows, network interfaces, and key GCP technologies currently used.

In the lower right corner of Figure  2 is an application created by HDC named Eureka. Eureka is a secure, scalable cloud computing and storage platform designed to enable advanced analytics on large or sensitive data sets without data leaving HDC’s secure cloud environment. Eureka instances can be created with a wide range of CPUs/TPUs, RAM memory, and persistent storage. Unlike standard cloud virtual machines (VMs), Eureka images have strict access controls designed to prevent data egress while allowing restricted access to core Internet software libraries and repositories. Extensive logging and auditing controls are embedded in the Eureka image. More details about Eureka are available at https://www.healthdatacompass.org/cloud-analytics-infrastructure.

**Figure 3.**
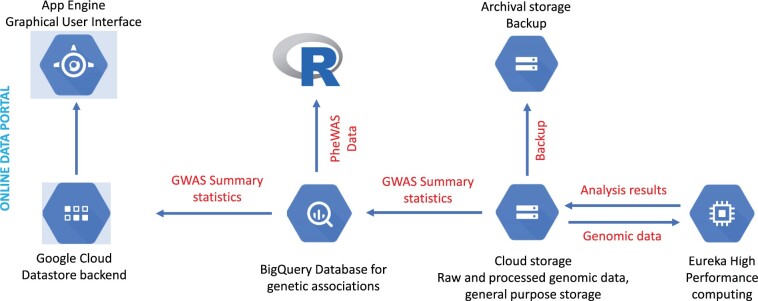
Data flows and key Google Cloud Platform (GCP) technologies used by the Translational Informatics Service (TIS). Although TIS uses fewer GCP technologies, TIS deploys more “forward-facing” (App Engine GUI, R Studio), high-performance computing (Eureka HPC), and cloud storage resources than does the RDW.

In August 2020, HDC’s parent organization, the Colorado Center for Personalized Medicine (CCPM), established the Translational Informatics Services (TIS). TIS provides computational services for large-scale genomic data, processes genotypes and genomic data into data sets useful for research and clinical use, implements standard and one-off bioinformatics pipelines, and supports partnerships between academia and industry. TIS migrated to a fully cloud-native infrastructure using HDC’s secure environment to store large files with raw and processed genetic data, accommodate diverse file formats, and leverage on-demand HPCCs to perform genome-wide association studies (GWAS), phenome-wide association studies (PheWAS), and other analyses. TIS core cloud components and data flows are shown in Figure  3.

Table  1 lists common RDW key performance measures illustrating the magnitude of data flows into and within HDC, current storage, data sources, and data requests/data delivery volumes. Also included are row counts from key clinical tables.

**Table 1.** Health data compass key performance indicators as of June 30, 2021

Health data compass key metrics	
Tables	790
Storage/clinical	16 TB
Storage/genomic	55TB
Extraction-transform-load jobs	2000+
Data sources	6 (3 internal; 3 external)
Unique persons	7.3M
Visits (all types)	51M
Conditions/Diagnoses (all types)	171M
Medications (ordered, administered, dispensed)	240M
Measurements (laboratory test)	1.3B
Observations (includes flowsheets)	6.6B
Clinical notes (all types)	210M
Custom data sets delivered	1286
Custom data marts/registries (local, national)	15
End-user applications	9

**Figure 4.**
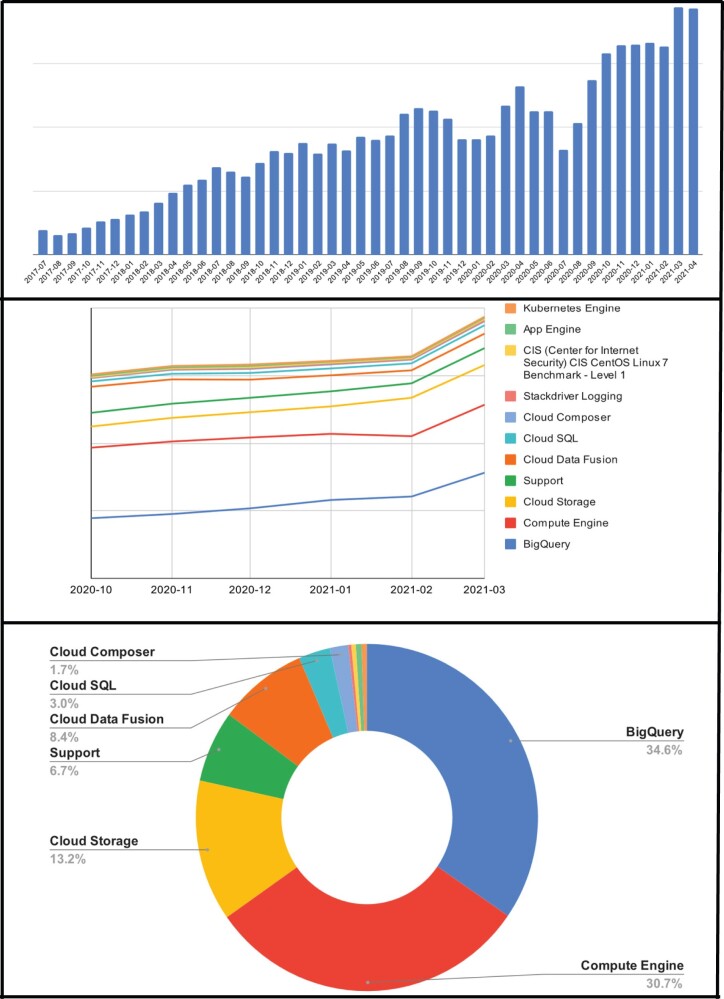
Top, Growth in Google Cloud Platform (GCP) total spend across all GCP services from July 2017. Middle, Growth of GCP monthly costs by specific GCP service October 2020–March 2021. Bottom, Proportion of charges across GCP services January–March 2021.

Figure  4 (top) displays growth in HDC’s total spend across all GCP products from July 2017 to March 2021. Figure  4 (center) is a cost breakdown by GCP services, and Figure  4 (bottom) illustrates the relative spend by GCP service from January to March 2021.

## DISCUSSION

It is daunting to move complex data flows, computations, and applications that support a wide range of translational research to any new environment. Differences in features and cost structures between on-premises and cloud infrastructures allow for unique opportunities and unexpected pitfalls. A simple “lift-and-shift” model that replicates on-premises hardware and software directly to cloud-hosted VMs may be most straightforward and comfortable for current teams to execute. But overlooking the cloud’s capability to instantly assign, alter, or release storage, computing, and networking resources or to provide entirely new services via simple application programming interface (API) changes can result in missed opportunities for cost savings or new innovations. Conversely, overlooking how new cloud products integrate into the existing cloud architecture or how usage charges accrue can quickly result in wasted resources, unexpected costs, unanticipated security and compliance risks, or conflicts with security controls, policies, or procedures.

Table  2 categorizes key areas of discovery during our RDW migration to GCP (details follow). Some issues are not unique to cloud-based implementations but are accentuated by the hyperdynamic technical advances in the public cloud marketplace. Other issues reflect platform limitations that existed when HDC architectural decisions were made. Given the continuous expansion of cloud capabilities, RDW teams responsible for cloud implementations must create processes and policies that anticipate a state of ongoing architectural and technical redesign while simultaneously supporting a large, complex, and heavily used operational RDW.

**Table 2.** Categories of underappreciated challenges that emerged during migration from on-premises to cloud data warehouse

Networking/Network security	Integration with enterprise networking System security plan/HIPAA Compliance Network access
Data engineering	Performance mismatch between source and cloud-based environments
Computation	Compute engines Managed services Cluster computing
Storage	Storage costs Tiered storage strategies Data provenance
Secure analytics	Cloud-based analytics Analytics repos vs security
Sandboxes/Public data	Sandboxes for low-barrier access Oversight Understanding public data sets
Innovation/Consulting services	Hyper-innovation/legacy architectures Development environments Consulting knowledge
Costs/utilization	Oversight and monitoring Leveraging cost-savings opportunities

### Networking and network security

Cloud tools and infrastructure listed by GCP as HIPAA compliant meet or exceed HIPAA security and privacy requirements. Institutional on-premises network security manages access control, threat detection, real-time alerting, compliance, and auditing. Existing on-premises security configurations have evolved over many years into deeply embedded infrastructure with approved policies tied to auditing and compliance procedures. HDC significantly underestimated the work necessary to translate the on-premises security environment (firewall, intrusion detection, logging, monitoring, alerting) using unfamiliar cloud-native security tools and the effort to integrate the new GCP security capabilities with the deployed institutional network design and security tools.

The security officers of our stakeholder institutions agreed upon NIST 800-53a as our target security compliance framework, a decision that significantly impacted costs, resources, and timelines.^12^ As early adopters, Compass faced significant concerns about institutional risks associated with large-scale fully identified patient data in the cloud. Because of internal experience with NIST 800-53a from participation in the National Children’s Study, the decision to implement NIST 800-53a controls helped accelerate acceptance. However, NIST 800-53a is a complicated, costly compliance framework to both implement and maintain. It is not strictly required to achieve sufficient technical security for HIPAA compliance. Specific security threats, the systems and processes that address each threat, and monitoring procedures to ensure compliance with the proposed solutions are contained in a Systems Security Plan (SSP). Security guidance documents for HIPAA, NIST 800-53 and HITRUST list hundreds of mandatory or recommended system and network security threats that require explicit implemented controls and compliance oversight.^13–15^ HDC’s current SSP consists of approximately 140 “moderate” NIST 800-53 controls, approved by our stakeholders security officers. Changes to the SSP require high-level institutional technical, regulatory, and legal engagement and approval. Thus, the long list of GCP HIPAA compliant products belies an enormous amount of additional work to ensure that a product is deployed in compliance within institutional security policies.

In retrospect, CU-AMC and HDC jointly significantly understaffed this activity. We allocated only 0.5 FTE across all tasks associated with creating a new SSP, policies, implementation, auditing procedures, and tools for the initial years. Our current estimate is that a combined effort of 2.5 FTEs across numerous institutional groups (HDC, network security, regulatory, compliance, legal) is a more realistic estimate in a multi-institutional environment managing highly confidential clinical and genomic data.

Another early decision was to limit network access to high-security VMs that performed critical ETL functions (ETL VMs). ETL VMs have network access only to institutional source systems (eg, hospital electronic medical records systems) and HDC-specific GCP networks. However, limited network access conflicts with a fundamental design assumption incorporated into many GCP products. These products are designed to pull the most recent version of software or containers from Google-managed repositories at the time the tool is activated—code repositories that were not accessible to the ETL VMs. Therefore, GCP tools failed with standard deployment designs. While not an ideal solution, hard-coding firewall rules to allow access to specific IP addresses was required for these tools to work.

### Data engineering: source versus cloud performance mismatch

HDC’s primary clinical data sources are electronic health record systems that house data in traditional relational database systems (RDBMS). These databases are also used by operational reporting units who compete with HDC for the same resources. Resource limitation policies control access to these high-demand databases. Thus, despite HDC’s access to scalable cloud computing resources, the initial extraction and transfer into the cloud is wholly determined by the on-premises RDBMS resource allocation to HDC. HDC has devised multiple optimization strategies to enable extractions to complete within the allowed restrictions. Once within HDC’s environment, resource constraints are nonexistent.

A second performance issue was handling increased network volumes. Due to source data model limitations, full table pulls rather than incremental loads are required. For very large tables, existing routers became network bottlenecks, requiring upgrades to the network infrastructure. A redesigned network architecture moved more network functions and traffic to scalable cloud-based routers, minimizing the amount of traffic between on-premises and cloud servers. ETL redesigns using incremental data extraction based on transaction logs may greatly decrease the amount of data moving across networks.

### Computation: virtual machines and managed services

Modern IT architectures use virtual machines or containers to enable allocating resources dynamically. With fixed hardware, adding new VMs is a zero-sum competition addressed either by restricting resources or purchasing more hardware. Public cloud vendors remove this resource competition, replacing the fixed upfront costs of acquiring new hardware with the variable costs of using more cloud resources.

To comply with our SSP each VM or container requires HDC to configure security settings and manage patches and upgrades to the operating system and hosted applications. An alternative to VMs are managed services, which encompasses software-as-a-service (SaaS), platform-as-a-service (PaaS), and infrastructure-as-a-service (IaaS). A managed service provides capabilities to a customer on an as-needed basis. SaaS requires the least amount of HDC management; IaaS requires the most. All current HDC design decisions prioritize SaaS over PaaS and PaaS over IaaS.

In practice, minimizing security and management overhead through higher level managed services has had mixed results. Figure  4 (bottom) shows Google BigQuery (GBQ), GCP’s SaaS large-scale database to be the highest GCP cost. By using BigQuery, HDC personnel no longer spend time fine-tuning DBMS parameters or scheduling activities around resource constraints. Similarly, technical personnel no longer spend time optimizing one-time queries. HDC does not employ a database administrator (DBA) despite its massive size. More technical services are focused on higher value use of resources. Managed services costs are offset by increased programmer productivity and more end-user services.

Managed services can also scale according to needs. For example, the TIS team generated genome-wide association studies (GWAS) summary statistics for more than 1000 phenotypes to support phenome-wide exploration of genetic associations (PheWAS). Fifty-four billion summary statistics for genetic variant/phenotype associations were stored in GBQ. It was possible to establish this 5.3TB repository without competing with other HDC resources.

The dynamic HPCC hosted within HDC’s HIPAA-compliant cloud, called Eureka HPC, enables genomic analytics performed by TIS to be used with fully identified biobank and clinical phenotype data. Eureka HPCC uses inexpensive preemptible VMs, which are standard VMs but with the caveat that Google can deallocate with a few minutes notice. This extremely cost-effective model is now used to run 2 production GWAS pipelines that utilize between 4 and 60 CPUs. Eureka HPCC allows large one-time jobs to be executed using the same HPCC infrastructure. For example, TIS deployed a large Eureka HPCC to compute 1260 GWAS analyses for ∼34 000 genotyped Biobank participants. This ephemeral HPCC cost $8730 or $6.93 per phenotype.

Despite a strong preference for using SaaS or PaaS managed services over IaaS virtual machines, Figure  4 shows that GCP VMs (Compute Engines) are HDC’s second largest cost. HDC hosts approximately 300 VMs. The majority of VM images are Eureka analytic engines which are created and terminated as-needed by end-users. Additionally, most data engineering development projects require 3 environments—development, test, and production—which multiplies the number of VMs. Other VMs host applications that only run on dedicated VMs such as OHDSI ATLAS,^16^ sandbox projects (described below), and the reluctance of some GCP SaaS vendors to sign Business Associate Agreements (BAA), forcing HDC to host the application.

### Storage

Access to essentially limitless storage eliminates a zero-sum competition for disk space. Cloud storage can use multiple geographical regions to ensure “5-9s” (99.999%) availability, a performance level that would be cost-prohibitive for a single institution. Backups are automatic with multiregion designs.

Because storage is inexpensive, HDC tends to keep everything. However limitless storage has downsides. An infrastructure with hundreds of users often results in duplication of the same or very similar data with little ability to reconstruct the chain of transformations. Archives and refreshes of these duplications can accumulate significant storage costs. It is difficult to know which data sets are in active use versus which can be archived or deleted. Even users have trouble keeping abreast their various data resources.

Since launching TIS, storage of large files with raw and processed genetic data in multiple file formats has highlighted the need to implement tiered storage to reduce costs. However, defining a tiered storage strategy that maximizes data availability while minimizing storage costs has been more challenging than envisioned. Data stored in high-latency tiers can be very cost-effective. However, the cost of migrating data from high-latency tiers back into online storage is expensive. Moving data between storage tiers only once or twice can obliterate the original cost savings. Uncertainty about data reuse has caused HDC to be cautious about using high-latency storage. Given the tendency of investigators to reanalyze old data with new hypotheses or tools, the amount of data deemed truly safe to put into cold storage has been surprisingly small.

### Secure analytics

Eureka is an analytics platform based on a high-security version of CentOS (Linux) designed to enable advanced analytics on large, PHI-containing data sets within HDC’s HIPAA compliant environment. Eureka enables users to scale both CPU and storage capacity to meet their analytic needs. Eureka costs are charged to the user based on cloud resources consumed. End-users control costs by “right-sizing” resources and turning off Eureka instances when not in use. Eureka instances can be deleted when no longer needed. Currently at Version 3.0, HDC has deployed approximately 100 Eureka instances.

Due to network security concerns, Eureka Version 1.0 blocked direct internet access. Eureka users were unable to pull directly from software repositories, like GitHub, to assemble packages or to update software. To offer more flexibility, Eureka 3.0 contains a growing safelist of public web resources to which a user can request time-limited access (60 minutes). The safe-list includes 9 major repository sites for data science, such as CRAN, Anaconda, PyPI, GitHub, and Bioconductor. Eureka continues to grow and evolve in response to user feedback.

### Sandboxes/public data sets

To enable broader access to GCP resources, HDC established lower security sandboxes where de-identified or synthetic data, such as Synthea^17^ and MIMIC^18^ can be made available and accessed directly along with the tools and capabilities of GCP. Sandboxes are used to “kick the tires” of new tools or standard VM- or container-based applications, such as OHDSI ATLAS^16^ and the University of Washington Leaf,^19^ to explore functionality and determine the value and effort required to integrate into a more secure environment. Cloud-based sandboxes do not compete with computational or storage resources used by existing projects.

However, ensuring users do not misuse sandbox environments by uploading sensitive data obtained outside HDC oversight is a challenge. Newer tools, such as GCP’s Data Loss Prevention, which scans data sets for sensitive information, may detect sensitive data in sandbox databases. In addition, there are no automated tools to determine when a sandbox is no longer needed other than examining when it was last assessed.

All public cloud vendors make a wide range of public and commercial data sets available for querying on their platforms. Google Marketplace currently lists 216 data sets available in BigQuery (https://console.cloud.google.com/marketplace/browse?filter=solution-type:dataset&pli = 1), including 43 data sets labeled as healthcare specific. The richness of readily available data resources has been a double-edged sword. Given scalable resources and easy availability, accessing these resources is trivial within the HDC platform. Our current challenges are understanding the strengths and weaknesses of each data source, what types of problems are best addressed by each resource, and how to query the data tables which limits our ability to leverage these resources. Thus, zero or minimal access costs have not translated into high or novel utilization.

### Innovation/consulting services

The speed and magnitude of new functionality in the cloud marketplace is daunting for HDC cloud architects to evaluate the utility of new offerings. In today’s cloud ecosystems, implementations are almost instantaneously legacy designs. The hyper-innovation of the cloud enables previously unattainable capabilities to become available simply via a new set of APIs. Determining what offering is a distraction versus a transformative opportunity takes time and carefully planned tests within sandboxes that replicate the existing architecture for head-to-head comparisons.

Once a new technology is deemed sufficiently promising to incorporate into production pipelines, extensive institutional review processes to comply with HDC’s SSP must be completed, including creating design documentation, risk analyses and assessments, and updating the SSP system boundaries. This requirement is not different from approval processes required for implementing new on-premises systems. However, the substantial personnel time across multiple institutional entities before production implementation extends the time between a new innovation and its availability in HDC. In the meantime, new product releases continue to occur, resulting in an sense of always falling behind to rapid-fire innovations.

Similarly, the rapid evolution of cloud-based technologies makes it difficult for internal architects and external consultants to obtain the deep experience with the leading-edge tools and technologies to leverage new capabilities. Overall, our experiences with consultants have been disappointing who tend to bring previous experiences with “out-of-the-box” designs. Few have experienced healthcare settings; none have implemented complex GCP-based solutions in a large-scale clinical research environment. The anticipated efficiencies of outside cloud expertise have been negated by prolonged knowledge transfer—the time and effort local resources consume educating consulting personnel on the nuances of our environment. When we have skipped extensive technical on-boarding by GCP technical members, initial implementations have not worked. HDC has learned how to better engage with external experts to ensure that local architectural features are highlighted from the beginning.

### Costs/utilization

Cloud-based resources are usually charged on a pay-as-you-use basis. Services can be turned on as needed, but also can be inadvertently kept active when not in use. Many services use different metrics to determine usage charges. BigQuery charges are based on data rows queried; Google Cloud Storage charges are based on size, access tier, and regions; Google Compute Engines are based on availability (pre-emptible), CPU, permanent storage needs, operating system, and uptime. Other services charge per-API call, per licensed user, or as a percent of other system charges. As the number of cloud services used by HDC has grown, aggregating and summarizing cloud charges has required more internal financial resources than anticipated. However, without careful oversight, unnecessary consumption-based costs can grow insidiously. For example, HDC did a comprehensive inventory of unused BigQuery data sets, virtual machines, and cloud storage. The resulting purge reduced monthly charges by approximately 20%.

New cloud capabilities also open new cost savings strategies as long as these opportunities are recognized, incorporated into daily practice, and displace more expensive practices. For example, because of the lack of a data orchestration tool, HDC’s ETL pipelines were initially constructed using a large Windows-based VM. The per-minute charge for this dedicated VM was high and it was used continuously for 3–4 days. A recent redesign uses a new data orchestration managed service that dynamically instantiates an array of inexpensive pre-emptible virtual machines which terminate in hours. The cost difference between the 2 ETL designs is significant, but cost savings were only realized after the new design was analyzed, approved, and implemented and the old design was retired.

## CONCLUSION

RDWs have become mission-critical strategic assets for advancing data-driven discoveries and next-generation clinical care. Given the explosive size and diversity of data in RDWs and the complexity of the data science now being applied to these data, traditional architectural designs are being displaced by cloud-based solutions. But the migration from traditional on-premises hardware and software is not as simple as moving the same tools and processes into a cloud-based environment. Public cloud vendors offer a tremendous array of new capabilities and access to resources on an as-needed basis, enabling innovation at scales and speeds not previously possible. At the same time, leveraging and managing this dynamic environment raises unique issues or accentuates similar issues seen in traditional settings.

HDC made an early decision to move to a fully-cloud RDW. At that time, it was the first significant foray into patient data management on a public cloud for HDC’s participating institutions. It also was the first enterprise-scale health data warehouse on GCP. HDC has never regretted this decision.

## FUNDING

This work was supported by the National Center for Advancing Translational Sciences (NCATS) grant number UL1 TR002535 to the Colorado Clinical and Translational Sciences Institute. Contents are the authors’ sole responsibility and do not necessarily represent official NIH views. Funds also provided by UCHealth, Childrens Hospital Colorado, and the University of Colorado.

## AUTHOR CONTRIBUTIONS

MGK, MJA, and NP developed the initial drafts of the manuscript. JYM, AKY, and NR provided detailed content related to governance, architecture, and infrastructure, respectively. All authors reviewed and approved the submitted manuscript and have agreed to be accountable for its contents.
